# Triglyceride-glucose index and the progression of chronic obstructive pulmonary disease, panvascular disease, and multimorbidity: a prospective cohort study

**DOI:** 10.3389/fcvm.2026.1855099

**Published:** 2026-08-26

**Authors:** Shuo Zhang, Xiaotian Li, Jiajing Li, Siwei Xie, Zongbin Wang, Zhijie Zheng, Jie Huang, Shuduo Zhou

**Affiliations:** 1 Department of Emergency, Peking University First Hospital, Beijing, China; 2 Department of Clinical Epidemiology, Peking University First Hospital, Beijing, China; 3 School of Health Humanities, Peking University, Beijing, China; 4 School of Public Health, Peking University, Beijing, China; 5 Johns Hopkins Bloomberg School of Public Health, Baltimore, MD, United States; 6 Department of Global Health, Peking University School of Public Health, Beijing, China; 7 School of Public Health and Emergency Management, Southern University of Science and Technology, Shenzhen, Guangdong, China

**Keywords:** chronic obstructive pulmonary disease, longitudinal progression, multimorbidity, panvascular disease, triglyceride-glucose index

## Abstract

**Objective:**

Chronic obstructive pulmonary disease (COPD) and panvascular disease (PD) are often driven by shared risk factors. We aimed to investigate the longitudinal association of triglyceride-glucose (TyG) index with incidence of COPD, PD, and their multimorbidity.

**Methods:**

Our study used data from the UK Biobank, a population-based prospective study. Cox proportional hazards regression was used to evaluate the association between TyG index and the risk of incident COPD, panvascular disease, and their Multimorbidity. Multistate models (MSMs) were used to evaluate the longitudinal progression from healthy to incident COPD or PD, and ultimately to multimorbidity, across TyG index quartiles.

**Results:**

Among the 296,818 participants included in the final analysis, the TyG index was associated with higher incidence of COPD and PD. Each 1-SD increase in TyG index was associated with a 9% higher risk of PD (HR 1.09, 95% CI 1.05–1.12). These associations were more pronounced among females. In multistate analyses, compared with the lowest quartile, women in the highest TyG quartile had greater risks of progression from baseline to COPD (HR 1.22, 95% CI 1.04–1.43), baseline to PD (HR 1.29, 95% CI 1.19–1.39), COPD to multimorbidity (HR 2.11, 95% CI 1.32–3.37), and PD to multimorbidity (HR 3.67, 95% CI 2.00–6.72). For males, a higher TyG index was protective against COPD incidence (HR 0.79, 95% CI 0.68–0.93) but increased PD risk (HR 1.18, 95% CI 1.09–1.26), whereas no significant associations were found for other transitions.

**Conclusions:**

Elevated TyG index was associated with incident COPD and PD, while the association with multimorbidity was attenuated after full adjustment in the overall cohort. In multistate analyses, stronger and more consistent associations were observed in women, particularly for progression from single disease to multimorbidity. The TyG index may serve as a metabolic indicator that helps identify women at elevated risk and may inform targeted prevention strategies.

## 1 Introduction

Chronic obstructive pulmonary disease (COPD) is the third leading cause of death worldwide and imposes a substantial economic burden, equivalent to 0.111% (0.085–0.141) of global GDP annually (1). Panvascular diseases (PD), a new concept proposed in recent years, refers to a series of vascular diseases, represented by coronary artery disease, ischaemic stroke, and peripheral artery disease (2), and accounts for 49.1% of coronary heart disease deaths and 35.2% of stroke deaths (3). By viewing these conditions as systemic manifestations of atherosclerosis, panvascular medicine emphasizes integrated prevention strategies rather than organ-specific approaches (2). COPD and PD often coexist due to shared risk factors (4), including smoking, inflammation, and oxidative stress, and their concurrence is associated with poorer prognosis and higher disease burden (5).

Insulin resistance (IR) has been proposed as a key biological link between COPD and PD (6, 7). Beyond lifestyle risk factors, IR may contribute to systemic inflammation, oxidative stress, and endothelial dysfunction, thereby accelerating pulmonary and vascular damage. Growing evidence has shown positive associations between IR and cardiovascular outcomes, including coronary heart disease, ischaemic stroke, and peripheral artery disease, across general and high-risk populations (8–10). Despite growing evidence, few studies have examined PD as an integrated entity or investigated the role of the IR in COPD–PD multimorbidity. Moreover, almost no research has investigated the longitudinal progression from baseline health to single disease and ultimately to multimorbidity—a critical omission, given that understanding this sequential evolution is fundamental to early risk stratification, timely intervention, and the prevention of irreversible disease accumulation. In addition, existing studies have predominantly focused on Asian populations, with limited data from large-scale European cohorts (11).

Although the hyperinsulinaemic–euglycaemic clamp remains the gold standard for assessing IR, its operational complexity precludes large-scale application. Unlike Homeostatic model assessment for insulin resistance (HOMA-IR) and the quantitative insulin sensitivity check index (QUICKI), which rely on fasting insulin, the TyG index is calculated solely from routine fasting glucose and triglycerides (11)—standard, low-cost tests available in most health panels. This makes the TyG index a practical, cost-effective surrogate for population screening and primary care settings where insulin measurement is not feasible.

To address the above gaps, we aimed to investigate the association of TyG index with the incidence of COPD, PD, and their multimorbidity, in a large European population from the UK Biobank. We further sought to explore the role of the TyG index in longitudinal disease progression from baseline health to single disease and ultimately to multimorbidity, with particular attention to potential sex-specific differences.

## 2 Materials and methods

### 2.1 Study design and population

**Figure 1:**
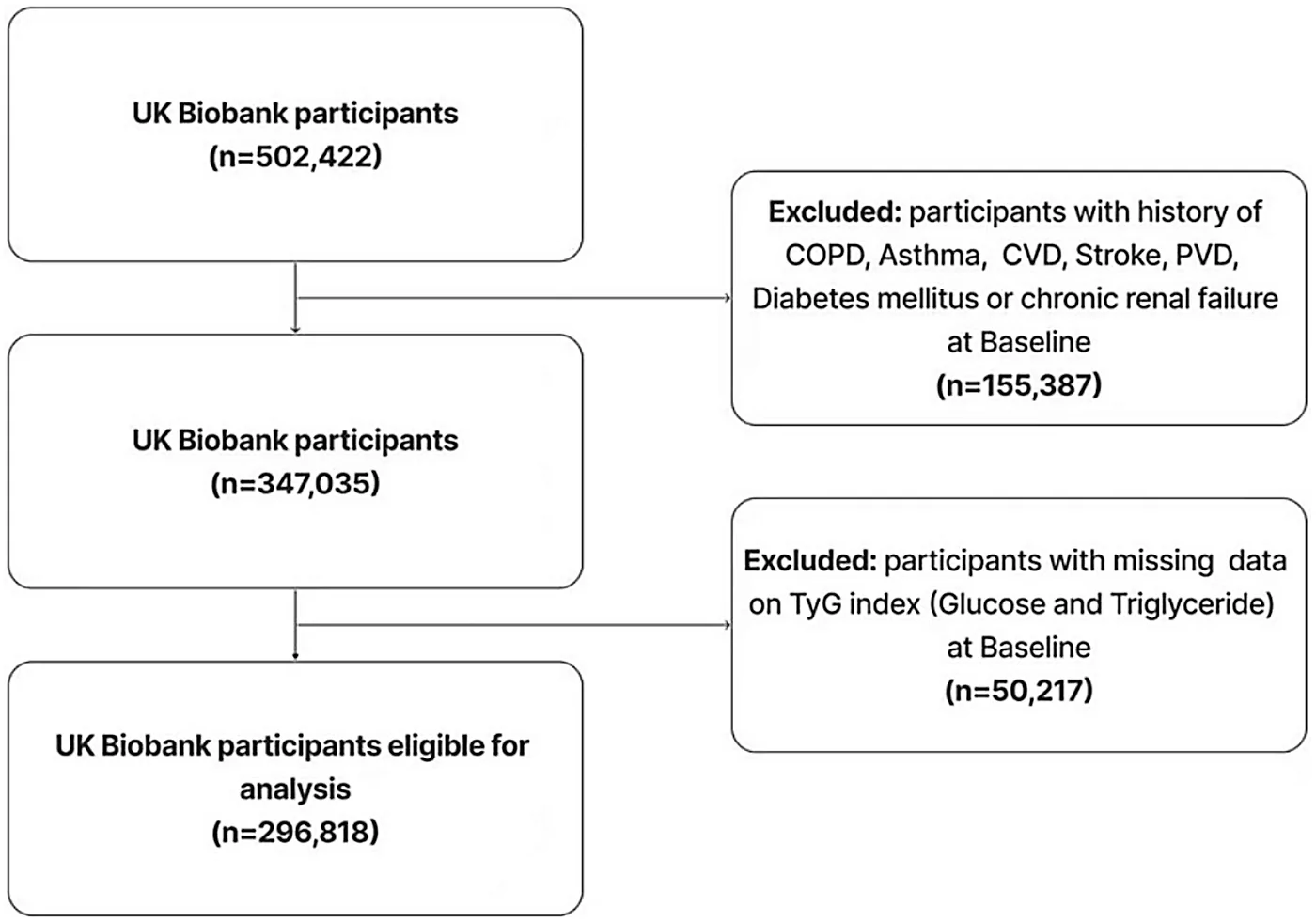
Flowchart showing participant selection for a UK Biobank study: 502,422 participants initially, 155,387 excluded for medical history, 50,217 excluded for missing TyG data, leaving 296,818 eligible for analysis. COPD, chronic obstructive pulmonary disease; CVD, Cardiovascular Disease; PVD, Peripheral Vascular Disease; TyG index, triglyceride-glucose index.

The UK Biobank is a population-based prospective cohort study, recruiting over 500,000 residents at 22 sites across England, Wales, and Scotland from 2006 to 2010 (12). In this study, we excluded participants with a history of either chronic obstructive pulmonary disease (COPD), asthma, cardiovascular disease (CVD), stroke, peripheral vascular disease (PVD), diabetes mellitus, or chronic renal failure at baseline (*n* = 155,387). Patients with missing data on examination results of TyG (*n* = 50,217) at baseline were also excluded. Ultimately, a total of 296,818 participants were included in this study (Figure 1).

This study was conducted according to the Declaration of Helsinki. The generic ethical approval was obtained by UK Biobank from the NHS National Research Ethics Service (approval letter dated June 17th 2011, Ref 11/NW/0382). All participants provided written informed consent to participate in the UK Biobank. The present study was carried out under Application Number 66137.

### 2.2 Data collection

Demographic information including age, sex, Townsend Deprivation Index(TDI), smoking status, drinking status, and physical activity level [<150 or ≥150 min/week, based on the total time spent in moderate physical activity each week (13)], were collected at baseline assessment. The TDI is a composite measure of socioeconomic deprivation, including four equally weighted variables: unemployment, overcrowded household, non-car ownership, and non-home ownership, with higher scores representing lower socioeconomic status (14). The Omron HEM-7015IT digital blood pressure monitor was used to measure the systolic and diastolic blood pressure. Body mass index (BMI) was calculated as weight in kilograms (kg) divided by height in meters squared (m^2^). Participants underwent blood sampling at baseline, and the process of sampling and biochemical analysis has been validated (15). At the central laboratory, the UK Biobank staff used the AU5800 (Beckman Coulter, CA, USA) to measure the serum concentration of glucose, triglycerides (TG), low density lipoprotein cholesterol (LDL-C), C-reactive protein (CRP), and uric acid. Glycated hemoglobin (HbA1c) was measured using the Variant II Turbo analyzer (Bio-Rad, CA, USA) through high-performance liquid chromatography. The estimated glomerular filtration rate (eGFR) was calculated using the 2021 CKD-EPI creatinine-cystatin C equation (16). The TyG index was calculated using the formula: ln [triglycerides (mg/dL)  ×  glucose (mg/dL)/2] (8).

### 2.3 Definition of outcome

The primary outcomes of this study were incident COPD, incident panvascular disease (PD) and multimorbidity. PD was operationally defined as major atherosclerotic vascular disease involving the coronary, cerebral, or peripheral arterial bed, including cardiovascular disease (CVD), stroke, or peripheral vascular disease (PVD). Multimorbidity was defined as the co-occurrence of incident COPD and PD. Unless otherwise stated, participant follow-up started at enrollment in the UK Biobank and was censored on October 21, 2021, on the date of loss to follow-up, or on the onset date of each outcome. COPD and PD incident events were identified through death registries, primary care records, hospital inpatient data, and self-reported diagnoses. The onset date of COPD or PD was defined as the date of first reported COPD or PD. The onset date of multimorbidity was defined as the date of reported COPD or the date of reported PD, whichever came late. Incident COPD was defined by the 10th revision of the International Classification of Diseases (ICD-10) codes J41-J44. Incident CVD was defined by ICD-10 codes I20-I25. Incident stroke was defined by ICD-10 codes I60-I64. Incident PVD was defined by ICD-10 codes I65-I66, I70, and I74.

### 2.4 Statistical analyses

Continuous variables were expressed as mean [Standard Deviation (SD)] and categorical variables as frequency (percentage). We stratified participants into four groups according to the quartiles of the TyG index at baseline. Trends across quartiles were tested by the generalized linear regression analysis for continuous variables and the Cochran-Armitage trend chi-square test for categorical variables, respectively.

The incidence of each defined outcome (COPD, PD or multimorbidity) in different TyG index quartiles was compared by log-rank test. Hazard ratios (HRs) and 95% confidence intervals (95%CIs) for each defined outcome, across TyG index quartiles and per 1-SD increment in TyG index, were estimated using Cox proportional hazard regression models. The proportional hazards assumption for the Cox models were assessed using Schoenfeld residuals and global tests. The TyG index quartiles were incorporated into the Cox model as a continuous variable to test the *P* values for linear trends across the quartiles of the TyG index. Univariable model and multivariable-adjusted models were presented: model 1 adjusted for none, model 2 adjusted for age (years), sex (male or female), and TDI, and model 3 further adjusted for physical activity (<150 min/week or ≥150 min/week), smoking status (current, previous, or never), drinking status (current, previous, or never), body mass index (kg/m^2^), systolic blood pressure (mmHg), diastolic blood pressure (mmHg), uric acid (mg/dL), C-reactive protein (mg/L), glycated hemoglobin (mmol/mol), low-density lipoprotein cholesterol (mg/dL) and estimated glomerular filtration rate(mL/min/1.73 m^2^). To examine potential multicollinearity, we calculated the variance inflation factor (VIF) for each independent variable. A VIF threshold of > 5 was prespecified to define concerning collinearity.

**Figure 2:**
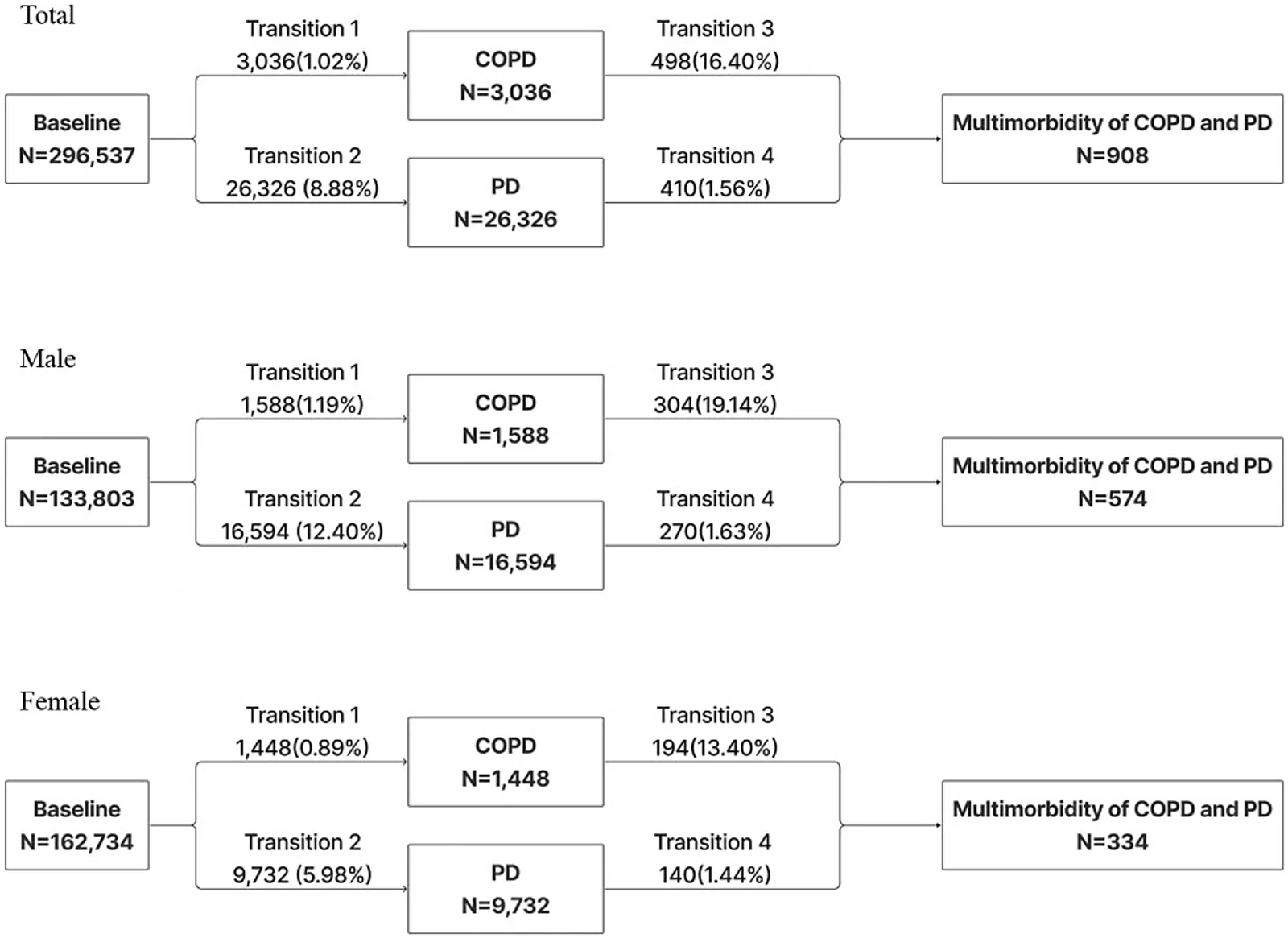
Flowchart diagram showing incidence and transitions between baseline, chronic obstructive pulmonary disease (COPD), Parkinson's disease (PD), and their multimorbidity in a cohort of 296,537, stratified by total, male, and female groups. Each path displays numbers and percentages transitioning from baseline to COPD or PD, then to multimorbidity. Males and females are analyzed separately, illustrating differences in disease progressions and multimorbidity rates, with final multimorbidity cases being 908 total, 574 male, and 334 female.

We used multistate models (MSMs) to evaluate the association between TyG index and longitudinal progression from healthy to incident COPD or PD, and ultimately to multimorbidity. The hazard ratios and 95% confidence intervals of higher quartiles in relation to the lowest quartiles were calculated. Four transitions were considered in this study (Figure 2): (1) transition 1: baseline to incident COPD, (2)transition 2: baseline to incident PD, (3) transition 3: COPD to multimorbidity, (4) transition 4: PD to multimorbidity. Three models applied in the Cox regression analysis were also employed in the multistate models. We excluded 281 participants, leaving 296,537 participants in the analysis, because we could not ascertain the temporal sequence of disease occurrences if a participant was diagnosed with COPD and PD on the same date. Furthermore, we conducted sex-stratified multistate model analyses, including 133,803 male and 162,734 female participants, to explore potential sex differences in the observed associations. The restricted cubic splines (RCS) were fitted with four knots placed at the 5th, 35th, 65th, and 95th percentiles to determine whether there was a nonlinear dose-response relationship of the TyG index with the risk of each defined outcome in the three models applied in the Cox regression analysis.

We conducted the sensitivity analysis to test the robustness of the current study. We excluded participants who developed the target event (COPD, PD, or multimorbidity of COPD and PD) or loss to follow-up within 2 follow-up years to check for reverse causation. Additionally, the same Cox analysis was performed in the participants whose BMI was within the range of normal weight (<25 kg/m^2^). Moreover, to eliminate the influence of COVID-19, participants who developed the target event (COPD, PD, or multimorbidity of COPD and PD) after January 1, 2020 were also excluded. Finally, we excluded haemorrhagic stroke from the PD definition to examine the robustness of our findings under alternative case definitions.

For subgroup analysis of the association between the TyG index quartiles and each defined outcome, the data were stratified by age (<60 or ≥60), sex (male or female), BMI [normal weight (<25 kg/m^2^), overweight (25–29.9 kg/m^2^) or obese (≥30 kg/m^2^)], physical activity (<150 min/week or ≥150 min/week), smoking status (never, previous or current), and drinking status (never, previous or current). Model 3 in the Cox regression analysis was applied for subgroup analysis.

In our study, the primary analyses were Cox regressions examining the associations between the TyG index and incident COPD, PD, and multimorbidity; the multistate models served as supportive analyses; and sex-stratified, subgroup, and sensitivity analyses were considered exploratory or robustness checks. The proportion of missing covariate data was between 0.07%–25.03%, and the missing covariate data were assigned by multiple imputation with the Markov chain Monte Carlo method. A significance level of two-tailed *P* < 0.05 was used to indicate statistical significance. All analyses were conducted using R version 4.4.1 (http://www.R-project.org, The R Foundation).

### 2.5 Patient and public involvement

Participating patients and the public were not involved in the design, or conduct, or reporting, or dissemination plans of this study.

## 3 Results

### 3.1 Characteristics of the participants

Baseline characteristics of the included and excluded participants are summarized in Supplementary Table S1. A total of 296,818 participants were included in the final analysis. The mean age was 56.3 (SD 8.1) years, 162,813 (54.9%) were women, and the mean Townsend Deprivation Index (TDI) score was −1.40 (SD 3.03). The TyG index cut-off values for Quartiles 1–4 were 6.37–8.30, 8.30–8.67, 8.67–9.06, and 9.06–12.14, respectively. Detailed baseline characteristics of the study population, along with comparisons across TyG quartiles, are presented in Table 1.

**Table 1:** Characteristics of the study population according to the TyG index quartiles.

Characteristics	TyG index					P trend
	Total	Quartile1 6.37∼8.30	Quartile2 8.30∼8.67	Quartile3 8.67∼9.06	Quartile4 9.06∼12.14	
N	296,818	74,204	74,204	74,204	74,204	
Female (%)	162,813 (54.9)	50,636 (68.2)	44,231 (59.6)	38,015 (51.2)	29,931 (40.3)	<0.001
Age at baseline, year	56.3 (8.1)	54 (8.2)	56.6 (8.0)	57.4 (7.8)	57.3 (7.8)	0.13
Townsend deprivation index	−1.4 (3.0)	−1.4 (3.1)	−1.5 (3.0)	−1.5 (3.0)	−1.3 (3.1)	<0.001
Physical activity, min/week						
≥150	24,379 (8.2)	6,374 (8.6)	6,775 (9.1)	7,124 (9.5)	6,760 (9.1)	<0.001
<150	272,439 (91.8)	67,829 (91.4)	67,429 (90.9)	67,080 (90.5)	67,444 (90.9)	<0.001
Smoking status (%)						
Current	30,894 (10.4)	6,384 (8.6)	7,079 (9.6)	7,869 (10.6)	9,562 (12.9)	<0.001
Previous	99,758 (33.6)	22,665 (30.5)	24,258 (32.7)	25,705 (34.6)	27,130 (36.6)	<0.001
Never	166,166 (56.0)	45,156 (60.9)	42,866 (57.8)	40,630 (54.8)	37,514 (50.6)	<0.001
Drinking status (%)						
Current	274,819 (92.6)	69,121 (93.1)	68,949 (92.9)	68,556 (92.4)	68,193 (91.9)	<0.001
Previous	9,451 (3.2)	2,184 (2.9)	2,243 (3.1)	2,400 (3.2)	2,623 (3.5)	<0.001
Never	12,548 (4.2)	2,900 (3.9)	3,012 (4.1)	3,247 (4.4)	3,389 (4.6)	<0.001
Body mass index, kg/m²	27.2 (4.6)	25.0 (3.9)	26.6 (4.4)	27.9 (4.5)	29.2 (4.6)	<0.001
Systolic blood pressure, mmHg	140 (21.8)	134 (19.5)	140 (19.8)	143 (19.6)	145 (19.3)	<0.001
Diastolic blood pressure, mmHg	82.6 (11.3)	79.1 (10.5)	81.7 (10.6)	83.3 (10.5)	84.7 (10.5)	<0.001
Glycated hemoglobin, mmol/mol	36.1 (7.2)	34.1 (4.2)	34.9 (4.2)	35.7 (4.7)	37.6 (8.3)	<0.001
C-reactive protein, mg/L	2.47 (4.1)	1.86 (4.0)	2.33 (4.2)	2.69 (4.3)	2.97 (4.0)	<0.001
Uric acid, mg/dL	5.15 (1.3)	4.56 (1.2)	4.96 (1.2)	5.31 (1.3)	5.78 (1.3)	<0.001
Low-density lipoprotein cholesterol, mg/dL	140 (32.8)	126 (27.9)	138 (30.3)	146 (32.3)	150 (35.1)	<0.001
Glucose, mmol/L	5.06 (1.1)	4.73 (0.5)	4.92 (0.6)	5.04 (0.7)	5.54 (1.7)	<0.001
Triglycerides, mmol/L	1.73 (1.0)	0.831 (0.2)	1.26 (0.2)	1.78(0.3)	3.06(1.0)	<0.001
eGFR, mL/min/1.73 m²	92.8(17.3)	94.4(16.8)	92.3(16.7)	91.8(17.1)	92.7(18.5)	<0.001

Continuous variables are expressed as mean (standard deviation); Categorical variables are expressed as numbers (percentage).

TyG, triglyceride-glucose; eGFR, estimated glomerular filtration rate.

In the longitudinal progression analysis, 296,537 participants were eligible at baseline after excluding those with simultaneous diagnoses of COPD and PD. The median follow-up was 12.0 years (IQR 11.7–13.4), comprising 133,803 men and 162,734 women. Overall, 3,036 (1.02%) participants developed COPD first, of whom 498 (16.40%) subsequently progressed to multimorbidity. In parallel, 26,326 (8.88%) participants developed PD first, and 410 (1.56%) later developed multimorbidity (Figure 2).

### 3.2 Prospective associations of the TyG index and risk of COPD, PD, and their multimorbidity

In fully adjusted Cox regression models, higher TyG index was associated with increased risk of incident COPD and PD (Table 2). Compared with the lowest quartile, the HRs (95% CIs) for Quartiles 2–4 were 1.06 (0.96–1.18), 1.11 (1.00–1.23), and 1.12 (1.00–1.25) for COPD, and 1.07 (1.02–1.11), 1.12 (1.07–1.17), and 1.22 (1.14–1.30) for PD. Each 1-SD increase in TyG index corresponded to a 4% higher risk of COPD (HR 1.04, 95% CI 0.99–1.08) and a 9% higher risk of PD (HR 1.09, 95% CI 1.05–1.12).

**Table 2:** Prospective associations between the TyG index and risk of COPD, PD, and the multimorbidity of COPD and PD in the UK biobank.

Variable	TyG index				P trend	Per 1 SD increase in log TyG values
	Quartile 1	Quartile 2	Quartile 3	Quartile 4		
Median	8.07	8.49	8.85	9.35		
COPD						
No. of cases/person-years	647/938,427	870/933,846	1,035/932,237	1,175/932,547	<0.001	
Incidence rate (per 1,000 person-years)	0.69	0.93	1.11	1.26		
Model 1	1.00	1.35 (1.22, 1.49)	1.60 (1.45, 1.77)	1.82 (1.66, 2.01)	<0.001	1.24 (1.20, 1.28)
Model 2	1.00	1.10 (0.99, 1.22)	1.21 (1.10, 1.34)	1.31 (1.19, 1.44)	<0.001	1.11 (1.08, 1.15)
Model 3	1.00	1.06 (0.96, 1.18)	1.11 (1.00, 1.23)	1.12 (1.00, 1.25)	0.04	1.04 (0.99, 1.08)
PD						
No. of cases/person-years	4,255/919,583	6,029/905,148	7,437/895,494	9,387/883,339	<0.001	
Incidence rate (per 1,000 person-years)	4.63	6.66	8.30	10.63		
Model 1	1.00	1.44 (1.38, 1.50)	1.79 (1.73, 1.86)	2.30 (2.22, 2.38)	<0.001	1.36 (1.34, 1.37)
Model 2	1.00	1.17 (1.12,1.21)	1.32 (1.27,1.37)	1.60 (1.54,1.66)	<0.001	1.21 (1.19,1.22)
Model 3	1.00	1.07 (1.02,1.11)	1.12 (1.07,1.17)	1.22 (1.14,1.30)	<0.001	1.09 (1.05,1.12)
Multimorbidity						
No. of cases/person-years	168/943,956	264/940,532	340/939,719	417/940,685	<0.001	
Incidence rate (per 1,000 person-years)	0.18	0.28	0.36	0.44		
Model 1	1.00	1.57 (1.30, 1.91)	2.03 (1.69, 2.44)	2.49 (2.08, 2.98)	<0.001	1.37 (1.30, 1.45)
Model 2	1.00	1.23 (1.02, 1.50)	1.43 (1.18, 1.72)	1.62 (1.35, 1.94)	<0.001	1.20 (1.13, 1.27)
Model 3	1.00	1.14 (0.93, 1.38)	1.20 (0.99, 1.45)	1.20 (0.98, 1.46)	0.11	1.05(0.98, 1.13)

Data are presented as HR (95%CI) unless otherwise stated. The TyG index was divided into 4 parts according to quartiles, and the boundaries were 6.37∼, 8.30∼, 8.67∼, 9.06∼12.14. Model 1was an unadjusted model.

Model 2 was adjusted for age, sex (male or female), and Townsend Deprivation Index. Model 3 was additionally adjusted for physical activity (<150 or ≥150 min/week), smoking status (current, previous, or never), drinking status (current, previous, or never), body mass index (kg/m²), systolic blood pressure (mmHg), diastolic blood pressure (mmHg), uric acid (mg/dL), C-reactive protein (mg/L), glycated hemoglobin(mmol/mol), low-density lipoprotein cholesterol (mg/dL) and estimated glomerular filtration rate (mL/min/1.73 m²). TyG, triglyceride-glucose; HR, hazard ratio; CI, confidence interval; COPD, chronic obstructive pulmonary disease; PD, panvascular disease; Multimorbidity, multimorbidity of COPD and PD.

**Figure 3:**
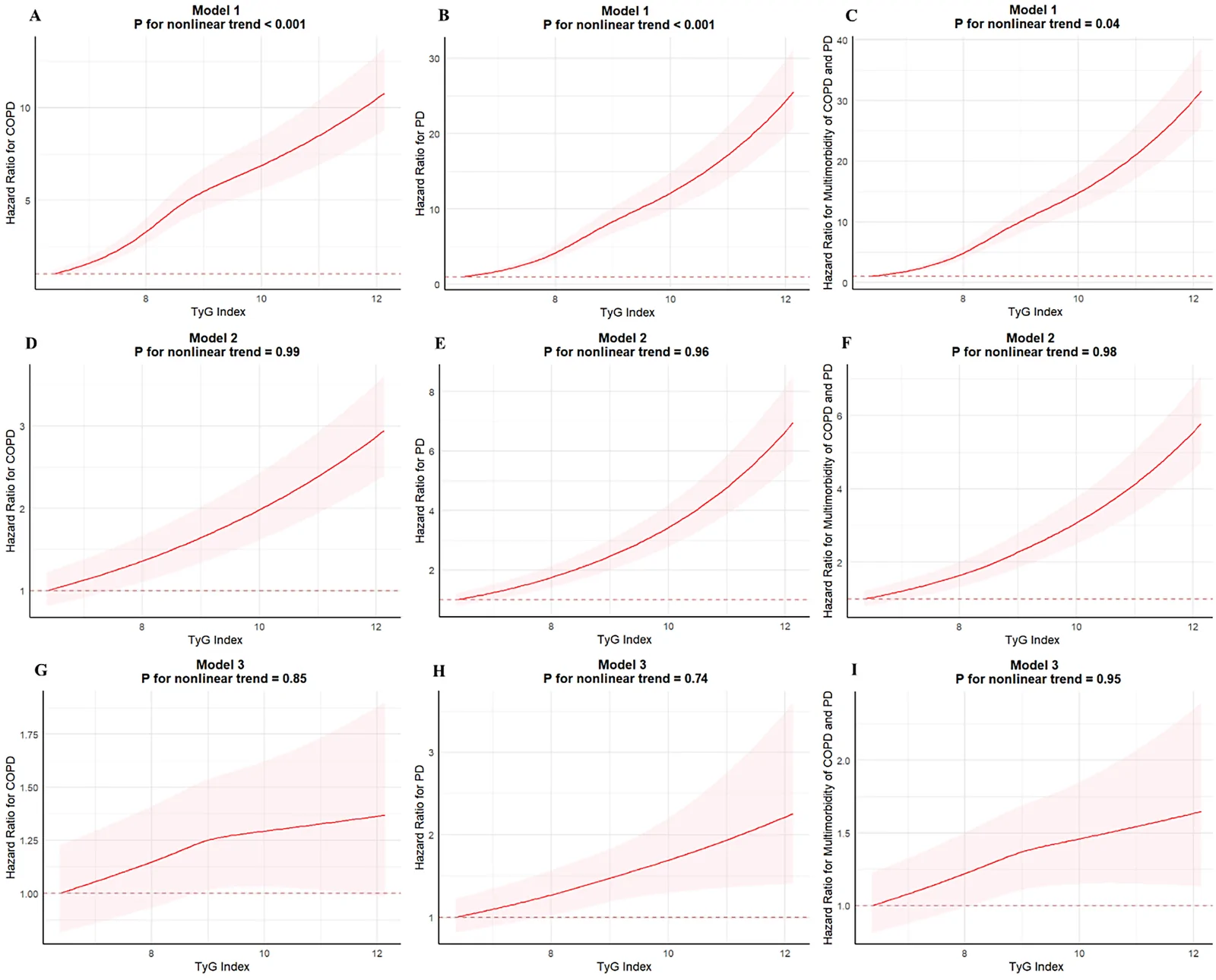
Hazard ratios and 95% confidence intervals for COPD, PD, and multimorbidity of COPD and PD associated with the TyG index. (A-C) Associations in Model 1 for COPD, PD, and multimorbidity of COPD and PD, respectively; (D-F) associations in Model 2 for COPD, PD, and multimorbidity of COPD and PD, respectively; (G-I) associations in Model 3 for COPD, PD, and multimorbidity of COPD and PD, respectively. COPD, chronic obstructive pulmonary disease; PD, panvascular disease; TyG index, triglyceride-glucose index.

For multimorbidity, positive associations were observed in age- and sex-adjusted analyses but became non-significant after full covariate adjustment (HRs for Quartiles 2–4: 1.14 [0.93–1.38], 1.20 [0.99–1.45], and 1.20 [0.98–1.46]). Restricted cubic spline analyses showed no evidence of non-linearity association of TyG index with risks of COPD, PD, and multimorbidity across the exposure range (Figure 3).

### 3.3 Longitudinal associations between TyG index and progression to COPD, PD, and their multimorbidity

In multistate models, the TyG index was significantly associated with the transition from baseline to PD in the overall population (Quartile 4 vs. Quartile 1: HR 1.24, 95% CI 1.16–1.32; Table 3). No associations were observed for transitions from baseline to COPD or from single disease to multimorbidity after full adjustment (Table 3; Supplementary Table S2.1).

**Table 3:** Multistate model to assess associations of TyG index with different transitions in Figure 2.

Group	TyG index	Baseline to COPD		Baseline to PD		COPD to Multimorbidity		PD to Multimorbidity	
		HR (95%CI)	P	HR (95%CI)	P	HR (95%CI)	P	HR (95%CI)	P
Total	Quartile 1	1		1		1		1	
	Quartile 2	0.99 (0.89, 1.11)	0.90	1.07 (1.03, 1.11)	0.001	1.31 (0.97, 1.77)	0.08	1.03 (0.74, 1.43)	0.86
	Quartile 3	0.98 (0.88, 1.09)	0.67	1.13 (1.08, 1.18)	<0.001	1.36 (1.02, 1.82)	0.04	1.21 (0.88, 1.64)	0.24
	Quartile 4	0.92 (0.81, 1.03)	0.14	1.24 (1.16, 1.32)	<0.001	1.31 (0.99, 1.75)	0.06	1.32 (0.98, 1.79)	0.07
Male	Quartile 1	1		1		1		1	
	Quartile 2	0.86 (0.74, 1.01)	0.07	1.05 (1.00, 1.11)	0.07	1.01 (0.69, 1.49)	0.95	0.89 (0.61, 1.30)	0.55
	Quartile 3	0.89 (0.77, 1.04)	0.14	1.08 (1.02, 1.14)	0.006	1.18 (0.82, 1.69)	0.37	0.75 (0.51, 1.08)	0.12
	Quartile 4	0.79 (0.68, 0.93)	0.004	1.18 (1.09, 1.26)	<0.001	0.97 (0.68, 1.39)	0.88	0.79 (0.55, 1.12)	0.18
Female	Quartile 1	1		1		1		1	
	Quartile 2	1.15 (0.98, 1.34)	0.08	1.07 (1.01, 1.14)	0.03	1.84 (1.15, 2.95)	0.01	1.39 (0.70, 2.76)	0.35
	Quartile 3	1.12 (0.96, 1.31)	0.15	1.17 (1.10, 1.24)	<0.001	1.62 (1.00, 2.61)	0.05	2.99 (1.62, 5.52)	<0.001
	Quartile 4	1.22 (1.04, 1.43)	0.02	1.29 (1.19, 1.39)	<0.001	2.11 (1.32, 3.37)	0.002	3.67(2.00, 6.72)	<0.001

Data are presented as HR (95%CI) unless otherwise stated. The TyG index was divided into 4 parts according to quartiles, and the boundaries were 6.37∼, 8.30∼, 8.67∼, 9.06∼12.14 for the total population, 6.37∼, 8.44∼, 8.81∼,9.20∼12.14 for male participants and 6.60∼, 8.21∼, 8.55∼, 8.93∼11.91 for female participants. Results were calculated based on Model 3. Model 3 was adjusted for age, sex (male or female), Townsend Deprivation Index, physical activity(<150 or ≥150 min/week), smoking status (current, previous, or never), drinking status (current, previous, or never), body mass index (kg/m²), systolic blood pressure(mmHg), diastolic blood pressure (mmHg), uric acid (mg/dL), C-reactive protein (mg/L), glycated hemoglobin(mmol/mol), low-density lipoprotein cholesterol(mg/dL) and estimated glomerular filtration rate (mL/min/1.73 m²).

TyG, triglyceride-glucose; HR, hazard ratio; CI, confidence interval; COPD, chronic obstructive pulmonary disease; PD, panvascular disease; Multimorbidity, multimorbidity of COPD and PD.

Among men, a higher TyG index showed divergent associations with disease transitions (Table 3; Supplementary Table S2.2): it was protective against COPD incidence from baseline (HR 0.79, 95% CI 0.68–0.93) but increased PD risk from baseline (HR 1.18, 95% CI 1.09–1.26), whereas no significant associations were found for other transitions. In contrast, among women, elevated TyG index was consistently associated with higher risks across all stages of disease progression: baseline to COPD (HR 1.22, 95% CI 1.04–1.43), baseline to PD (HR 1.29, 95% CI 1.19–1.39), COPD to multimorbidity (HR 2.11, 95% CI 1.32–3.37), and PD to multimorbidity (HR 3.67, 95% CI 2.00–6.72; Table 3; Supplementary Table S2.3). The magnitude of associations was stronger for transitions from single diseases to multimorbidity than for transitions from baseline to single diseases.

### 3.4 Subgroup and sensitivity analyses

Subgroup analyses are shown in Supplementary Tables S2.1–S2.3. For COPD, significant interactions were observed by age, sex, and BMI. Among participants younger than 60 years or with normal weight, higher TyG index was associated with greater COPD risk. The association was particularly pronounced in women, with HRs (95% CIs) of 1.26 (1.07–1.50), 1.23 (1.04–1.46), and 1.39 (1.17–1.66) for Quartiles 2–4 compared with Quartile 1 (Supplementary Table S3.1), whereas no associations were detected in men (Supplementary Table S3.1). For PD, interactions were observed across age, sex, BMI, and smoking status, with stronger associations in participants aged <60 years, women, and never-smokers (Supplementary Table S3.2). For multimorbidity, effect modification was identified by age, sex, and BMI, with significant associations restricted to women (Supplementary Table S3.3).

In sensitivity analyses, exclusion of participants who developed outcomes or were lost to follow-up within the first two years yielded consistent results for PD and multimorbidity (Supplementary Table S4.1), but attenuated the association with COPD (Supplementary Table S4.1). Restricting the analysis to participants with normal body weight produced similar estimates (Supplementary Table S4.2) and excluding events occurring after January 1, 2020 (excluding the impact of COVID-19) or excluding haemorrhagic stroke from the PD definition did not materially change the results (Supplementary Table S4.3–S4.4).

To formally evaluate effect modification by sex, we included a sex-by-TyG interaction term in both the Cox and multistate models. In the Cox regression (Model 3), the interaction was significant for COPD, PD, and multimorbidity (all P interaction < 0.001). In the multistate models, significant interactions were observed across all transitions (baseline→COPD: P interaction < 0.001; baseline→PD: Pinteraction = 0.010; COPD→multimorbidity: P interaction = 0.006; PD→multimorbidity: P interaction < 0.001). These findings indicate that the associations were significantly modified by sex.

Before conducting multivariate regression analyses, we assessed multicollinearity among the included variables using variance inflation factors (VIF). The results indicated no substantial multicollinearity (all VIF values < 5), with the detailed diagnostic data provided in Supplementary Table 5.

## 4 Discussion

In this large prospective cohort study, we found that a higher TyG index was associated with increased risk of COPD, PD, while the association with multimorbidity was attenuated after full adjustment in the overall cohort. Restricted cubic spline analyses showed no evidence of non-linearity association of TyG index with risks of COPD, PD, and multimorbidity across the exposure range. In multistate analyses, higher TyG index was significantly associated with disease progression across all transitions in women. These associations were evident at each stage of the disease progression, and were particularly pronounced during the transition from single diseases to multimorbidity. In contrast, a higher TyG index was protective against COPD incidence from baseline but increased PD risk from baseline among males. To the best of our knowledge, this study was the first comprehensive investigation of the relationship between the TyG index and the longitudinal progression of COPD, PD, and their Multimorbidity.

Limited research has focused on the correlation between the TyG index and incident COPD. A cross-sectional analysis of NHANES 1999–2012 first reported that each unit increase in the TyG index was associated with a 21% higher risk of chronic bronchitis, whereas no association was observed for emphysema (17). This finding may indirectly support our observation of a stronger association in women, who tend to exhibit more pronounced small airway disease, characterized by smaller luminal dimensions and thicker airway walls, and relatively less emphysema than men (18). In the Malmö Preventive Project, Zaigham et al. showed that TyG index predicted incident COPD in women and lifelong never-smokers over 31 years of follow-up (19). Using NHANES 1999–2018 data, Guo et al. further confirmed positive associations between TyG index and COPD prevalence in non-diabetic women, but not in men or individuals with diabetes (20). These findings align with our results in females, which additionally demonstrate that higher TyG index is associated with COPD incidence among European participants with normal body weight and those younger than 60 years. However, unlike in females, our study revealed higher TyG index was protective against COPD in males.

As a surrogate marker of insulin resistance, the TyG index may link to COPD through shared behavioral risks such as smoking and physical inactivity (6). Beyond these exposures, dyslipidaemia and hyperglycaemia, reflected by elevated TyG index, can trigger systemic inflammation and oxidative stress, activate intracellular signalling pathways, and promote the release of inflammatory mediators that impair pulmonary structure and function (6, 17, 21–23). Nevertheless, the underlying biological mechanisms remain insufficiently understood and warrant further investigation. Besides, the observed sex differences in the association between the TyG index and COPD outcomes warrant further investigation. Although the underlying mechanisms remain unclear, previous studies have proposed several potential pathways that may contribute to such differences. For instance, testosterone, which is markedly higher in males than females, has been associated with enhanced insulin sensitivity, increased lean mass, reduced subcutaneous fat, and lower inflammatory markers including CRP (24). These anabolic and anti-inflammatory effects have been suggested to mitigate the adverse metabolic consequences of insulin resistance, which could theoretically render a high TyG index less detrimental in males than in females. Additionally, given the well-established link between malnutrition and poor lung function (25), it has been hypothesized that a higher TyG index, potentially reflecting greater caloric intake and metabolic turnover, might confer relative protection against COPD in males. This notion is indirectly supported by a previous study reporting that overweight and obesity were associated with reduced COPD risk in males but not in females (25). However, it must be emphasized that none of these mechanisms were directly assessed in our study. Therefore, the above interpretations remain speculative and should be viewed as hypothesis-generating rather than confirmatory. Future studies with direct measurements of sex hormones, inflammatory biomarkers, body composition, and nutritional status are needed to elucidate the underlying mechanisms and to validate or refute these hypotheses.

While many studies have linked the TyG index to individual cardiovascular outcomes, few have assessed these conditions collectively or examined their multimorbidity with COPD (8–10, 26–28). Consistent with previous literature, our findings showed a positive association between the TyG index and the incidence of PD. We further observed that the association appeared to be stronger in women, yet sex differences have received limited attention and are rarely discussed in other studies. Previous research (29) has found that levels of total cholesterol and low-density lipoprotein (LDL) cholesterol are more influential in determining cardiovascular disease (CVD) risk in men. In contrast, elevated triglycerides and lipoprotein(a) levels, reduced high-density lipoprotein (HDL) cholesterol levels, and the presence of diabetes appear to be more strongly associated with CVD risk in women. The TyG index reflects the combined metabolic impact of elevated triglycerides and hyperglycemia (11). This synergistic effect may explain its stronger association with PD and multimorbidity observed in females. The protective effects of testosterone (24) detailed above also contribute to the less detrimental impact of elevated TyG index in males compared with females. A significant gap persists in understanding the sex-specific mechanisms, and further research is necessary to elucidate these differences and inform the development of more effective, sex-tailored therapeutic strategies (30).

Our findings revealed that the TyG index was strongly associated with female disease progression, particularly during the transition from single diseases to multimorbidity. This suggests that the TyG index may be more reflective of disease progression or clustering rather than the initiation of individual pathological processes. Such a distinction is critical for understanding the natural history of relating chronic disease development and for informing the development of targeted preventive and therapeutic interventions. Given that multimorbidity is linked to increased healthcare utilization, diminished quality of life, and elevated mortality risk (5),the TyG index, as a metabolic indicator, holds significant potential for enabling earlier and more effective intervention strategies. The recognition of the TyG index as a significant risk factor, particularly in women, carries important implications for public health and clinical practice. Specifically, there is a compelling need for targeted health education initiatives to raise awareness among women regarding the TyG index as a surrogate marker of insulin resistance and its association with increased risk of COPD, PD, and their multimorbidity. Moreover, it suggests that women may warrant closer evaluation in future studies and risk-stratified prevention frameworks and highlights the necessity for sex-specific screening strategies to facilitate early detection and effective disease management. Such strategies may contribute to reducing the burden of associated diseases and improving overall health outcomes in this population. Research has demonstrated that physical activity may reduce TyG levels (31, 32). Interventions aimed at improving insulin sensitivity, such as lifestyle modifications, weight management strategies, and pharmacological therapies, may hold promise for delaying or preventing the progression to multimorbidity in women at high risk (9). Future interventional studies are warranted to confirm the effects of the TyG index and to better elucidate the underlying mechanisms involved.

This study addresses a significant gap in the literature by comprehensively evaluating the correlation between the triglyceride-glucose index and the incidence of COPD, PD, and their multimorbidity in females, an area that has received limited attention in prior research. Nonetheless, limitations are worth noting. (1) Recall bias may arise from self-reported data, and adjustment for confounders (lung function, diet, medication, menopause, hormone therapy, and air pollution) was precluded by data unavailability. Excluding baseline CVD, diabetes, or asthma may have introduced collider bias. The TyG index was not adjustment for fasting duration or inter-center processing variability. Besides, event dates in routine records reflect date of ascertainment rather than true disease onset, some misclassification in temporal ordering is possible. COPD was ascertained via ICD-10 codes without spirometric confirmation, potentially under-ascertaining mild cases and misclassifying asthma or chronic bronchitis. Future studies with spirometry-confirmed COPD, medication-based definitions, standardized laboratory protocols, adjustment for more confounders, and documented fasting duration are warranted. (2) Owing to multiple testing, the sex-stratified, subgroup, and transition-specific results are hypothesis-generating and should be interpreted with caution. Death was treated as censoring rather than a competing event due to unavailable mortality dates, which may have biased estimated associations, especially among older participants with a higher likelihood of death during follow-up. Additionally, adjusting for potential mediators (e.g., BMI, inflammatory markers, triglycerides, and HbA1c) in fully adjusted models may attenuate effect estimates and obscure causal inferences. Future studies incorporating competing-risk analyses and formal mediation frameworks are needed to validate our findings. (3) Our analysis was restricted to the baseline TyG index, as repeated measurements during follow-up were unavailable, precluding evaluation of dynamic changes or cumulative exposure. Systematic comparisons with other IR surrogates (HOMA-IR, METS-IR, TyG-BMI, TyG-WC, TyG-WHtR, and remnant cholesterol) and external validation were not performed, and future studies are needed to establish whether the TyG index offers incremental value over these alternatives in this specific clinical context.

## 5 Conclusions

In conclusion, our study demonstrates that IR, as assessed by the TyG index, is positively associated with the longitudinal progression of COPD, PD, and multimorbidity in females. These associations were particularly pronounced during the transition from isolated diseases to comorbid conditions. Our findings highlight the TyG index may serve as a metabolic indicator that helps identify women at increased risk of developing these conditions, especially multimorbidity involving COPD and PD. This study emphasizes the need for sex-specific approaches in the prevention, diagnosis, and management of diseases in women and provides a foundation for future research in this critical area. Further research is warranted to elucidate the causal direction of this association and to more thoroughly investigate the underlying pathophysiological mechanisms.

## Data Availability

Publicly available datasets were analyzed in this study. This data can be found here: All data relevant to the study were acquired from the UK Biobank Resource. Data can be accessed through applications on UK Biobank website (https://www.ukbiobank.ac.uk/).
