# Supplementary material for: Triglyceride-glucose index and the progression of chronic obstructive pulmonary disease, panvascular disease, and multimorbidity: a prospective cohort study
Source: Front Cardiovasc Med. 2026 Aug 26;13:1855099. doi: 10.3389/fcvm.2026.1855099 (PMC13553825; doi:10.3389/fcvm.2026.1855099)
Supplement: Supplementary file 1 [file Datasheet1.docx]

**Supplementary Table 1** Baseline characteristics of included and excluded participants.

| **Characteristics** | **Excluded** | **Included** | **P value** | **SMD** |
| --- | --- | --- | --- | --- |
| N | 205,604 | 296,818 |  |  |
| Female (%) | 110,519 (53.8) | 162,813 (54.9) | <0.001 | 0.022 |
| Age at baseline, year | 56.87 (8.1) | 56.30 (8.1) | <0.001 | 0.07 |
| Townsend Deprivation Index | -1.14 (3.2) | -1.40 (3.0) | <0.001 | 0.086 |
| Physical activity, min/week | | | 0.838 | 0.001 |
| ≥150 | 14,863 (9.1) | 22,634 (9.1) | |  |
| <150 | 148,246 (90.9) | 225,230 (90.9) | |  |
| Smoking status(%) | |  | <0.001 | 0.063 |
| Current | 22,193 (10.8) | 30,770 (10.4) | |  |
| Previous | 73,648 (36.0) | 99,380 (33.5) | |  |
| Never | 107,903 (52.7) | 165,579 (55.8) | |  |
| Drinking status(%) | |  | <0.001 | 0.065 |
| Current | 185,823 (90.8) | 274,469 (92.5) | |  |
| Previous | 8,661 (4.2) | 9,434 (3.2) |  |  |
| Never | 9,857 (4.8) | 12,525 (4.2) | |  |
| Body mass index, kg/m^2^ | 27.79 (5.1) | 27.18 (4.6) | <0.001 | 0.126 |
| Systolic blood pressure, mmHg | 139.66 (19.6) | 139.80 (19.8) | 0.017 | 0.007 |
| Diastolic blood pressure, mmHg | 81.90 (10.7) | 82.41 (10.7) | <0.001 | 0.048 |
| Glycated hemoglobin, mmol/mol | 36.97 (8.1) | 35.57 (5.7) | <0.001 | 0.2 |
| C-reactive protein, mg/L | 2.83 (4.7) | 2.46 (4.1) | <0.001 | 0.083 |
| Uric acid, mg/dL | 5.27 (1.4) | 5.15 (1.3) | <0.001 | 0.082 |
| Low-density lipoprotein cholesterol, mg/dL | 133.68 (34.7) | 139.77 (32.8) | <0.001 | 0.18 |
| Glucose, mmol/L | 5.27 (1.6) | 5.06 (1.1) | <0.001 | 0.161 |
| Triglycerides, mmol/L | 1.78 (1.1) | 1.73 (1.0) | <0.001 | 0.043 |
| eGFR (mL/min/1.73m^2^) | 93.56 (15.6) | 95.61 (13.9) | <0.001 | 0.139 |

Continuous variables are expressed as mean (standard deviation); Categorical variables are expressed as

numbers (percentage). Abbreviations: SMD, Standardized Mean Difference; TyG, triglyceride-glucose; eGFR,

estimated glomerular filtration rate.

**Supplementary Table 2.1** Multistate model to assess associations of TyG index with different transitions in FIGURE 2 among 296,537 participants

| **Model** | **TyG index** | **Baseline to COPD** | | **Baseline to PD** | | **COPD to Multimorbidity** | | **PD to Multimorbidity** | |
| --- | --- | --- | --- | --- | --- | --- | --- | --- | --- |
|  |  | **HR (95%CI)** | **P** | **HR (95%CI)** | **P** | **HR (95%CI)** | **P** | **HR (95%CI)** | **P** |
| **Model 1** | **Quartile 1** | 1 |  | 1 |  | 1 |  | 1 |  |
|  | **Quartile 2** | 1.33(1.19, 1.48) | <0.001 | 1.43(1.37, 1.49) | <0.001 | 1.75(1.30, 2.36) | <0.001 | 1.38(0.99, 1.92) | 0.06 |
|  | **Quartile 3** | 1.54(1.38, 1.72) | <0.001 | 1.78(1.71, 1.85) | <0.001 | 2.15(1.61, 2.86) | <0.001 | 1.90(1.40, 2.59) | <0.001 |
|  | **Quartile 4** | 1.69(1.52, 1.88) | <0.001 | 2.29(2.20, 2.37) | <0.001 | 2.43(1.83, 3.22) | <0.001 | 2.44(1.81, 3.29) | <0.001 |
| **Model 2** | **Quartile 1** | 1 |  | 1 |  | 1 |  | 1 |  |
|  | **Quartile 2** | 1.08(0.96, 1.21) | 0.18 | 1.16(1.12, 1.21) | <0.001 | 1.42(1.06, 1.92) | 0.02 | 1.12(0.81, 1.56) | 0.50 |
|  | **Quartile 3** | 1.14(1.02, 1.27) | 0.02 | 1.32(1.27, 1.37) | <0.001 | 1.59(1.19, 2.12) | 0.002 | 1.41(1.03, 1.92) | 0.03 |
|  | **Quartile 4** | 1.19(1.07, 1.32) | 0.001 | 1.60(1.54, 1.66) | <0.001 | 1.70(1.28, 2.26) | <0.001 | 1.71(1.27, 2.31) | <0.001 |
| **Model 3** | **Quartile 1** | 1 |  | 1 |  | 1 |  | 1 |  |
|  | **Quartile 2** | 0.99(0.89, 1.11) | 0.90 | 1.07(1.03, 1.11) | 0.001 | 1.31(0.97, 1.77) | 0.08 | 1.03(0.74, 1.43) | 0.86 |
|  | **Quartile 3** | 0.98(0.88, 1.09) | 0.67 | 1.13(1.08, 1.18) | <0.001 | 1.36(1.02, 1.82) | 0.04 | 1.21(0.88, 1.64) | 0.24 |
|  | **Quartile 4** | 0.92(0.81, 1.03) | 0.14 | 1.24(1.16, 1.32) | <0.001 | 1.31(0.99, 1.75) | 0.06 | 1.32(0.98, 1.79) | 0.07 |

Data are presented as HR (95%CI) unless otherwise stated. The TyG index was divided into 4 parts according

to quartiles, and the boundaries were 6.37~, 8.30~, 8.67~, 9.06~12.14. Model 1was an unadjusted model.

Model 2 was adjusted for age, gender (male or female), and Townsend Deprivation Index. Model 3 was

additionally adjusted for physical activity (<150 or ≥150 mins/week), smoking status (current, previous, or

never), drinking status (current, previous, or never), body mass index (kg/m2), systolic blood pressure

(mmHg), diastolic blood pressure (mmHg), uric acid (mg/dL), C-reactive protein (mg/L), glycated hemoglobin

(mmol/mol), low-density lipoprotein cholesterol (mg/dL) and estimated glomerular filtration rate(mL/min/1.73

m^2^). Abbreviations: TyG, triglyceride-glucose; HR, hazard ratio; CI, confidence interval; COPD, chronic

obstructive pulmonary disease; PD, panvascular disease; Multimorbidity, multimorbidity of COPD and PD.

| **Model** | **TyG index** | **Baseline to COPD** | | **Baseline to PD** | | **COPD to Multimorbidity** | | **PD to Multimorbidity** | |
| --- | --- | --- | --- | --- | --- | --- | --- | --- | --- |
|  |  | **HR (95%CI)** | **P** | **HR (95%CI)** | **P** | **HR (95%CI)** | **P** | **HR (95%CI)** | **P** |
| **Model 1** | **Quartile 1** | 1 |  | 1 |  | 1 |  | 1 |  |
|  | **Quartile 2** | 0.98(0.84, 1.15) | 0.84 | 1.20(1.14, 1.26) | <0.001 | 1.15(0.78, 1.70) | 0.48 | 1.02(0.70, 1.48) | 0.94 |
|  | **Quartile 3** | 1.09(0.94, 1.27) | 0.27 | 1.32(1.25, 1.38) | <0.001 | 1.44(1.00, 2.06) | 0.05 | 0.91(0.63, 1.32) | 0.61 |
|  | **Quartile 4** | 1.04(0.90, 1.21) | 0.57 | 1.54(1.47, 1.61) | <0.001 | 1.27(0.89, 1.82) | 0.18 | 1.03(0.73, 1.46) | 0.86 |
| **Model 2** | **Quartile 1** | 1 |  | 1 |  | 1 |  | 1 |  |
|  | **Quartile 2** | 0.93(0.79, 1.09) | 0.35 | 1.13(1.07, 1.19) | <0.001 | 1.08(0.74, 1.60) | 0.68 | 0.96(0.66, 1.39) | 0.81 |
|  | **Quartile 3** | 1.02(0.88, 1.19) | 0.78 | 1.24(1.17, 1.30) | <0.001 | 1.35(0.94, 1.94) | 0.10 | 0.85(0.59, 1.24) | 0.40 |
|  | **Quartile 4** | 1.01(0.87, 1.17) | 0.93 | 1.49(1.42, 1.56) | <0.001 | 1.23(0.86, 1.76) | 0.25 | 1.00(0.70, 1.41) | 0.98 |
| **Model 3** | **Quartile 1** | 1 |  | 1 |  | 1 |  | 1 |  |
|  | **Quartile 2** | 0.86(0.74, 1.01) | 0.07 | 1.05(1.00, 1.11) | 0.07 | 1.01(0.69, 1.49) | 0.95 | 0.89(0.61, 1.30) | 0.55 |
|  | **Quartile 3** | 0.89(0.77, 1.04) | 0.14 | 1.08(1.02, 1.14) | 0.006 | 1.18(0.82, 1.69) | 0.37 | 0.75(0.51, 1.08) | 0.12 |
|  | **Quartile 4** | 0.79(0.68, 0.93) | 0.004 | 1.18(1.09, 1.26) | <0.001 | 0.97(0.68, 1.39) | 0.88 | 0.79(0.55, 1.12) | 0.18 |

**Supplementary Table 2.2** Multistate model to assess associations of TyG index with different transitions in FIGURE 2 among 133,803 male participants

Data are presented as HR (95%CI) unless otherwise stated. The TyG index was divided into 4 parts according

to quartiles, and the boundaries were 6.37~, 8.44~,8.81~,9.20~12.14. Model 1was an unadjusted model.

Model 2 was adjusted for age, gender (male or female), and Townsend Deprivation Index. Model 3 was

additionally adjusted for physical activity (<150 or ≥150 mins/week), smoking status (current, previous, or

never), drinking status (current, previous, or never), body mass index (kg/m2), systolic blood pressure

(mmHg), diastolic blood pressure (mmHg), uric acid (mg/dL), C-reactive protein (mg/L), glycated hemoglobin

(mmol/mol), low-density lipoprotein cholesterol (mg/dL) and estimated glomerular filtration rate(mL/min/1.73

m^2^).Abbreviations: TyG, triglyceride-glucose; HR, hazard ratio; CI, confidence interval; COPD, chronic

obstructive pulmonary disease; PD, panvascular disease; Multimorbidity, multimorbidity of COPD and PD.

**Supplementary Table 2.3** Multistate model to assess associations of TyG index with different transitions in Figure 2 among 162,734 female participants

| **Model** | **TyG index** | **Baseline to COPD** | | **Baseline to PD** | | **COPD to Multimorbidity** | | **PD to Multimorbidity** | |
| --- | --- | --- | --- | --- | --- | --- | --- | --- | --- |
|  |  | **HR (95%CI)** | **P** | **HR (95%CI)** | **P** | **HR (95%CI)** | **P** | **HR (95%CI)** | **P** |
| **Model 1** | **Quartile 1** | 1 |  | 1 |  | 1 |  | 1 |  |
|  | **Quartile 2** | 1.60(1.37, 1.87) | <0.001 | 1.49(1.41, 1.59) | <0.001 | 2.57(1.60, 4.11) | <0.001 | 1.94(0.98, 3.85) | 0.06 |
|  | **Quartile 3** | 1.88(1.61, 2.19) | <0.001 | 1.95(1.84, 2.07) | <0.001 | 2.72(1.68, 4.38) | <0.001 | 5.02(2.72, 9.25) | <0.001 |
|  | **Quartile 4** | 2.42(2.08, 2.82) | <0.001 | 2.55(2.40, 2.70) | <0.001 | 4.20(2.64, 6.68) | <0.001 | 7.29(3.99, 13.33) | <0.001 |
| **Model 2** | **Quartile 1** | 1 |  | 1 |  | 1 |  | 1 |  |
|  | **Quartile 2** | 1.26(1.08, 1.47) | 0.003 | 1.18(1.11, 1.25) | <0.001 | 2.03(1.26, 3.25) | 0.003 | 1.53(0.77, 3.04) | 0.22 |
|  | **Quartile 3** | 1.34(1.15, 1.56) | <0.001 | 1.39(1.31, 1.48) | <0.001 | 1.94(1.20, 3.13) | 0.007 | 3.58(1.94, 6.60) | <0.001 |
|  | **Quartile 4** | 1.63(1.40, 1.90) | <0.001 | 1.72(1.62, 1.82) | <0.001 | 2.83(1.78, 4.50) | <0.001 | 4.91(2.69, 8.99) | <0.001 |
| **Model 3** | **Quartile 1** | 1 |  | 1 |  | 1 |  | 1 |  |
|  | **Quartile 2** | 1.15(0.98, 1.34) | 0.08 | 1.07(1.01, 1.14) | 0.03 | 1.84(1.15, 2.95) | 0.01 | 1.39(0.70, 2.76) | 0.35 |
|  | **Quartile 3** | 1.12(0.96, 1.31) | 0.15 | 1.17(1.10, 1.24) | <0.001 | 1.62(1.00, 2.61) | 0.05 | 2.99(1.62, 5.52) | <0.001 |
|  | **Quartile 4** | 1.22(1.04, 1.43) | 0.02 | 1.29(1.19, 1.39) | <0.001 | 2.11(1.32, 3.37) | 0.002 | 3.67(2.00, 6.72) | <0.001 |

Data are presented as HR (95%CI) unless otherwise stated. The TyG index was divided into 4 parts according

to quartiles, and the boundaries were 6.60~, 8.21~, 8.55~, 8.93~11.91. Model 1was an unadjusted model.

Model 2 was adjusted for age, gender (male or female), and Townsend Deprivation Index. Model 3 was

additionally adjusted for physical activity (<150 or ≥150 mins/week), smoking status (current, previous, or

never), drinking status (current, previous, or never), body mass index (kg/m2), systolic blood pressure

(mmHg), diastolic blood pressure (mmHg), uric acid (mg/dL), C-reactive protein (mg/L), glycated hemoglobin

(mmol/mol), low-density lipoprotein cholesterol (mg/dL) and estimated glomerular filtration rate(mL/min/1.73

m^2^). Abbreviations: TyG, triglyceride-glucose; HR, hazard ratio; CI, confidence interval; COPD, chronic

obstructive pulmonary disease; PD, panvascular disease; Multimorbidity, multimorbidity of COPD and PD.

**Supplementary Table 3.1** Stratified analyses of the association between TyG index and risk of COPD

| **Subgroup** | **TyG index** | | | |  |
| --- | --- | --- | --- | --- | --- |
|  | **Quartile 1** | **Quartile 2** | **Quartile3** | **Quartile 4** | **P for interaction** |
|  | **HR (95%CI)** | **HR (95%CI)** | **HR (95%CI)** | **HR (95%CI)** |  |
| Age |  |  |  |  | 0.02 |
| <60 | 1 | 0.98(0.81, 1.18) | 1.16(0.96, 1.40) | 1.21(1.00, 1.48) |  |
| ≥60 | 1 | 0.98(0.88, 1.10) | 1.00(0.89, 1.13) | 0.98(0.86, 1.11) |  |
| Gender |  |  |  |  | <0.001 |
| Male | 1 | 0.95(0.84, 1.08) | 0.94(0.82, 1.07) | 0.95(0.83, 1.09) |  |
| Female | 1 | 1.26(1.07, 1.50) | 1.23(1.04, 1.46) | 1.39(1.17, 1.66) |  |
| BMI |  |  |  |  | <0.001 |
| Normal weight | 1 | 0.99(0.82, 1.19) | 1.12(0.94, 1.35) | 1.24(1.03, 1.48) |  |
| Overweight | 1 | 0.99(0.84, 1.16) | 1.12(0.96, 1.32) | 1.12(0.94, 1.32) |  |
| Obese | 1 | 0.96(0.81, 1.16) | 1.02(0.85, 1.23) | 1.06(0.88, 1.27) |  |
| Physical activity |  |  |  |  | 0.49 |
| <150min/week | 1 | 1.04(0.93, 1.16) | 1.10(0.98, 1.23) | 1.11(0.98, 1.24) |  |
| ≥150min/week | 1 | 1.36(0.99, 1.88) | 1.32(0.92, 1.88) | 1.34(0.95, 1.87) |  |
| Smoking status |  |  |  |  | 0.07 |
| Never | 1 | 1.07(0.82, 1.38) | 1.18(0.91, 1.52) | 1.03(0.78, 1.36) |  |
| Previous | 1 | 1.00(0.85, 1.17) | 1.10(0.93, 1.29) | 1.22(1.03, 1.44) |  |
| Current | 1 | 1.03(0.90, 1.19) | 1.12(0.96, 1.29) | 1.00(0.85, 1.18) |  |
| Drinking status |  |  |  |  | 0.38 |
| Never | 1 | 1.01(0.60, 1.72) | 1.68(1.03, 2.75) | 1.36(0.80, 2.32) |  |
| Previous | 1 | 1.00(0.68, 1.47) | 1.04(0.71, 1.53) | 0.87(0.57, 1.32) |  |
| Current | 1 | 1.06(0.95, 1.18) | 1.09(0.97, 1.22) | 1.12(1.00, 1.26) |  |

Data are presented as HR (95%CI) unless otherwise stated. The TyG index was divided into 4 parts according

to quartiles, and the boundaries were 6.37~, 8.30~, 8.67~, 9.06~12.14.Results were calculated based on Model

3. Model 3 was adjusted for age, gender (male or female), and Townsend Deprivation Index, physical activity

(<150 or ≥150 mins/week), smoking status (current, previous, or never), drinking status (current, previous, or

never), body mass index (kg/m2), systolic blood pressure(mmHg), diastolic blood pressure (mmHg), uric acid

(mg/dL), C-reactive protein (mg/L), glycated hemoglobin(mmol/mol), low-density lipoprotein cholesterol

(mg/dL) and estimated glomerular filtration rate(mL/min/1.73m^2^).Abbreviations: TyG, triglyceride-glucose;

HR, hazard ratio; CI, confidence interval; COPD, chronic obstructive pulmonary disease; PD, panvascular

disease; Multimorbidity, multimorbidity of COPD and PD.

**Supplementary Table 3.2** Stratified analyses of the association between TyG index and risk of PD

| **Subgroup** | **TyG index** | | | |  |
| --- | --- | --- | --- | --- | --- |
|  | **Quartile 1** | **Quartile 2** | **Quartile 3** | **Quartile 4** | **P for interaction** |
|  | **HR (95%CI)** | **HR (95%CI)** | **HR (95%CI)** | **HR (95%CI)** |  |
| Age |  |  |  |  | <0.001 |
| <60 | 1 | 1.12(1.04, 1.20) | 1.20(1.12, 1.29) | 1.40(1.28, 1.53) |  |
| ≥60 | 1 | 1.03(0.99, 1.08) | 1.07(1.02, 1.12) | 1.12(1.05, 1.20) |  |
| Gender |  |  |  |  | 0.004 |
| Male | 1 | 1.06(1.01, 1.11) | 1.07(1.02, 1.13) | 1.17(1.09, 1.27) |  |
| Female | 1 | 1.05(0.98, 1.12) | 1.16(1.08, 1.24) | 1.28(1.19, 1.38) |  |
| BMI |  |  |  |  | <0.001 |
| Normal weight | 1 | 0.97(0.90, 1.05) | 1.09(1.01, 1.19) | 1.18(1.09, 1.28) |  |
| Overweight | 1 | 1.00(0.95, 1.06) | 1.08(1.02, 1.15) | 1.19(1.11, 1.27) |  |
| Obese | 1 | 1.05(0.98, 1.13) | 1.04(0.96, 1.12) | 1.13(1.01, 1.26) |  |
| Physical activity |  |  |  |  | 0.15 |
| <150min/week | 1 | 1.06(1.02, 1.11) | 1.11(1.07, 1.17) | 1.22(1.14, 1.31) |  |
| ≥150min/week | 1 | 1.10(0.97, 1.25) | 1.16(1.02, 1.32) | 1.19(1.03, 1.36) |  |
| Smoking status |  |  |  |  | 0.02 |
| Never | 1 | 1.06(1.00, 1.13) | 1.09(1.03, 1.16) | 1.23(1.14, 1.31) |  |
| Previous | 1 | 1.07(1.00, 1.14) | 1.10(1.03, 1.17) | 1.20(1.11, 1.30) |  |
| Current | 1 | 1.08(0.98, 1.19) | 1.12(1.01, 1.24) | 1.20(1.06, 1.35) |  |
| Drinking status |  |  |  |  | 0.29 |
| Never | 1 | 1.09(0.91, 1.30) | 1.10(0.92, 1.32) | 1.27(1.04, 1.55) |  |
| Previous | 1 | 1.07(0.89, 1.29) | 1.14(0.95, 1.38) | 1.19(0.97, 1.45) |  |
| Current | 1 | 1.06(1.02, 1.11) | 1.12(1.07, 1.17) | 1.22(1.15, 1.30) |  |

Data are presented as HR (95%CI) unless otherwise stated. The TyG index was divided into 4 parts according

to quartiles, and the boundaries were 6.37~, 8.30~, 8.67~, 9.06~12.14.Results were calculated based on Model

3. Model 3 was adjusted for age, gender (male or female), and Townsend Deprivation Index, physical activity

(<150 or ≥150 mins/week), smoking status (current, previous, or never), drinking status (current, previous, or

never), body mass index (kg/m2), systolic blood pressure(mmHg), diastolic blood pressure (mmHg), uric acid

(mg/dL), C-reactive protein (mg/L), glycated hemoglobin(mmol/mol), low-density lipoprotein cholesterol

(mg/dL) and estimated glomerular filtration rate(mL/min/1.73m^2^).Abbreviations: TyG, triglyceride-glucose;

HR, hazard ratio; CI, confidence interval; COPD, chronic obstructive pulmonary disease; PD, panvascular

disease; Multimorbidity, multimorbidity of COPD and PD.

**Supplementary Table 3.3** Stratified analyses of the association between TyG index and risk of multimorbidity of COPD and PD

| **Subgroup** | **TyG index quartiles** | | | |  |
| --- | --- | --- | --- | --- | --- |
|  | **Quartile 1** | **Quartile 2** | **Quartile 3** | **Quartile 4** | **P for interaction** |
|  | **HR (95%CI)** | **HR (95%CI)** | **HR (95%CI)** | **HR (95%CI)** |  |
| Age |  |  |  |  | 0.03 |
| <60 | 1 | 0.57(0.37, 0.86) | 0.95(0.65, 1.37) | 0.86(0.59, 1.27) |  |
| ≥60 | 1 | 1.20(0.98, 1.48) | 1.17(0.95, 1.44) | 1.18(0.95, 1.46) |  |
| Gender |  |  |  |  | <0.001 |
| Male | 1 | 1.04(0.84, 1.28) | 0.93(0.75, 1.16) | 0.98(0.78, 1.23) |  |
| Female | 1 | 1.54(1.02, 2.33) | 1.76(1.18, 2.63) | 2.00(1.34, 2.99) |  |
| BMI |  |  |  |  | 0.006 |
| Normal weight | 1 | 0.77(0.52, 1.13) | 1.19(0.84, 1.68) | 1.15(0.81, 1.64) |  |
| Overweight | 1 | 1.06(0.78, 1.45) | 1.24(0.92, 1.68) | 1.21(0.89, 1.66) |  |
| Obese | 1 | 1.23(0.91, 1.65) | 1.10(0.81, 1.48) | 1.17(0.87, 1.59) |  |
| Physical activity |  |  |  |  | 0.13 |
| <150min/week | 1 | 1.12(0.91, 1.39) | 1.19(0.96, 1.46) | 1.24(1.00, 1.53) |  |
| ≥150min/week | 1 | 1.74(0.81, 3.74) | 1.60(0.74, 3.49) | 1.26(0.62, 2.56) |  |
| Smoking status |  |  |  |  | 0.09 |
| Never | 1 | 1.37(0.81, 2.31) | 1.38(0.82, 2.32) | 1.12(0.65, 1.96) |  |
| Previous | 1 | 1.12(0.81, 1.53) | 1.26(0.93, 1.72) | 1.37(1.01, 1.87) |  |
| Current | 1 | 1.11(0.86, 1.44) | 1.08(0.83, 1.41) | 1.07(0.81, 1.42) |  |
| Drinking status |  |  |  |  | 0.29 |
| Never | 1 | 0.93(0.34, 2.54) | 1.89(0.78, 4.58) | 1.24(0.46, 3.31) |  |
| Previous | 1 | 2.16(0.93, 5.01) | 1.59(0.67, 3.78) | 2.11(0.89, 4.99) |  |
| Current | 1 | 1.11(0.90, 1.36) | 1.15(0.94, 1.41) | 1.16(0.94, 1.43) |  |

Data are presented as HR (95%CI) unless otherwise stated. The TyG index was divided into 4 parts according

to quartiles, and the boundaries were 6.37~, 8.30~, 8.67~, 9.06~12.14.Results were calculated based on Model

3. Model 3 was adjusted for age, gender (male or female), and Townsend Deprivation Index, physical activity

(<150 or ≥150 mins/week), smoking status (current, previous, or never), drinking status (current, previous, or

never), body mass index (kg/m2), systolic blood pressure(mmHg), diastolic blood pressure (mmHg), uric acid

(mg/dL), C-reactive protein (mg/L), glycated hemoglobin(mmol/mol), low-density lipoprotein cholesterol

(mg/dL) and estimated glomerular filtration rate(mL/min/1.73m^2^).Abbreviations: TyG, triglyceride-glucose;

HR, hazard ratio; CI, confidence interval; COPD, chronic obstructive pulmonary disease; PD, panvascular

disease; Multimorbidity, multimorbidity of COPD and PD.

**Supplementary Table 4.1** Sensitivity Analysis: Excluding Individuals who developed the target event (COPD, PD, or their multimorbidity) or lost to follow-up within the first 2 years

| **Variable** | **TyG index** | | | | **P trend** | **Per 1 SD increase in Log TyG values** |
| --- | --- | --- | --- | --- | --- | --- |
|  | **Quartile 1** | **Quartile 2** | **Quartile 3** | **Quartile 4** |  |  |
| **COPD** |  |  |  |  |  |  |
| Median | 8.07 | 8.49 | 8.85 | 9.35 |  |  |
| No. of cases/person-years | 587/937,903 | 778/933,711 | 931/932,229 | 1,055/932,672 | <0.001 |  |
| Model 1, HR (95%CI) | 1.00 | 1.33(1.19, 1.48) | 1.59(1.43, 1.76) | 1.80(1.63, 2.00) | <0.001 | 1.24(1.20, 1.28) |
| Model 2, HR (95%CI) | 1.00 | 1.09(0.98, 1.21) | 1.20(1.08, 1.34) | 1.30(1.18, 1.44) | <0.001 | 1.11(1.07, 1.15) |
| Model 3, HR (95%CI) | 1.00 | 1.05(0.94, 1.17) | 1.10(0.99, 1.23) | 1.10(0.98, 1.24) | 0.06 | 1.03(0.99, 1.08) |
| **PD** |  |  |  |  |  |  |
| Median | 8.07 | 8.49 | 8.85 | 9.34 |  |  |
| No. of cases/person-years | 3,745/914,393 | 5,270/902,903 | 6,478/895,702 | 8,152/886,923 | <0.001 |  |
| Model 1, HR (95%CI) | 1.00 | 1.43(1.37, 1.49) | 1.77(1.70, 1.84) | 2.26(2.17, 2.34) | <0.001 | 1.35(1.33, 1.37) |
| Model 2, HR (95%CI) | 1.00 | 1.16(1.11, 1.21) | 1.31(1.26, 1.36) | 1.58(1.51, 1.64) | <0.001 | 1.20(1.19, 1.22) |
| Model 3, HR (95%CI) | 1.00 | 1.06(1.01, 1.10) | 1.11(1.06, 1.16) | 1.20(1.12, 1.28) | <0.001 | 1.08(1.04, 1.12) |
| **Multimorbidity** |  |  |  |  |  |  |
| Median | 8.07 | 8.49 | 8.85 | 9.35 |  |  |
| No. of cases/person-years | 156/943,845 | 247/940,491 | 322/939,680 | 392/940,681 | <0.001 |  |
| Model 1, HR (95%CI) | 1.00 | 1.59(1.30, 1.94) | 2.07(1.71, 2.50) | 2.52(2.09, 3.03) | <0.001 | 1.37(1.30, 1.45) |
| Model 2, HR (95%CI) | 1.00 | 1.25(1.02, 1.52) | 1.46(1.20, 1.77) | 1.64(1.36, 1.98) | <0.001 | 1.20(1.13, 1.27) |
| Model 3, HR (95%CI) | 1.00 | 1.14(0.93, 1.40) | 1.21(0.99, 1.48) | 1.19(0.97, 1.47) | 0.12 | 1.04(0.97, 1.12) |

Data are presented as HR (95%CI) unless otherwise stated. The number of participants was 296,344 for

COPD, 293,258 for PD, and 296,648 for Multimorbidity after excluding individuals who developed disease or

lost to follow-up within the first 2 years. The TyG index was divided into 4 parts according to quartiles, and

the boundaries were 6.37~, 8.30~, 8.67~, 9.06~12.14. Model 1was an unadjusted model. Model 2 was adjusted

for age, gender (male or female), and Townsend Deprivation Index. Model 3 was additionally adjusted for

physical activity (<150 or ≥150 mins/week), smoking status (current, previous, or never), drinking status

(current, previous, or never), body mass index (kg/m2), systolic blood pressure (mmHg), diastolic blood

pressure (mmHg), uric acid (mg/dL), C-reactive protein (mg/L), glycated hemoglobin (mmol/mol), low-density

lipoprotein cholesterol (mg/dL) and estimated glomerular filtration rate(mL/min/1.73m^2^).Abbreviations: TyG,

triglyceride-glucose; HR, hazard ratio; CI, confidence interval; COPD, chronic obstructive pulmonary disease;

PD, panvascular disease; Multimorbidity, multimorbidity of COPD and PD.

**Supplementary Table 4.2** Sensitivity Analysis: Conducted Among Individuals with Normal BMI

| **Variable** | **TyG index** | | | | **P trend** | **Per 1 SD increase in Log TyG values** |
| --- | --- | --- | --- | --- | --- | --- |
|  | **Quartile 1** | **Quartile 2** | **Quartile 3** | **Quartile 4** |  |  |
| Median | 7.91 | 8.26 | 8.57 | 9.02 |  |  |
| **COPD** |  |  |  |  |  |  |
| No. of cases/person-years | 205/325,281 | 257/322,945 | 333/322,061 | 437/321,472 | <0.001 |  |
| Model 1, HR (95%CI) | 1.00 | 1.25(1.04, 1.51) | 1.63(1.37, 1.94) | 2.14(1.81, 2.53) | <0.001 | 1.32(1.25, 1.39) |
| Model 2, HR (95%CI) | 1.00 | 1.02(0.85, 1.23) | 1.17(0.98, 1.39) | 1.35(1.14, 1.60) | <0.001 | 1.13(1.06, 1.19) |
| Model 3, HR (95%CI) | 1.00 | 0.99(0.82, 1.19) | 1.12(0.94, 1.35) | 1.24(1.03, 1.48) | 0.005 | 1.08(1.01, 1.15) |
| **PD** |  |  |  |  |  |  |
| No. of cases/person-years | 1,076/321,085 | 1,348/317,355 | 1,781/314,686 | 2,338/310,793 | <0.001 |  |
| Model 1, HR (95%CI) | 1.00 | 1.27(1.17, 1.37) | 1.69(1.56, 1.82) | 2.24(2.09, 2.41) | <0.001 | 1.37(1.34, 1.40) |
| Model 2, HR (95%CI) | 1.00 | 1.02(0.94, 1.11) | 1.20(1.11, 1.30) | 1.39(1.29, 1.50) | <0.001 | 1.17(1.14, 1.20) |
| Model 3, HR (95%CI) | 1.00 | 0.97(0.90, 1.05) | 1.09(1.01, 1.19) | 1.18(1.09, 1.28) | <0.001 | 1.09(1.06, 1.12) |
| **Multimorbidity** |  |  |  |  |  |  |
| No. of cases/person-years | 54/327,103 | 55/325,136 | 99/324,584 | 124/324,638 | <0.001 |  |
| Model 1, HR (95%CI) | 1.00 | 1.02(0.70, 1.48) | 1.84(1.32, 2.56) | 2.30(1.67, 3.17) | <0.001 | 1.40(1.26, 1.55) |
| Model 2, HR (95%CI) | 1.00 | 0.80(0.54, 1.17) | 1.23(0.88, 1.72) | 1.27(0.91, 1.77) | 0.02 | 1.14(1.02, 1.28) |
| Model 3, HR (95%CI) | 1.00 | 0.77(0.52, 1.13) | 1.19(0.84, 1.68) | 1.15(0.81, 1.64) | 0.10 | 1.07(0.95, 1.21) |

Data are presented as HR(95%CI) unless otherwise stated. The medians of the TyG quartiles, incidence density,

and Model 1 are based on the unimputed original data due to the inconsistent sample sizes of the imputed

databases. Model 1was an unadjusted model. Model 2 was adjusted for age, gender (male or female), and

Townsend Deprivation Index. Model 3 was additionally adjusted for physical activity (<150 or ≥150

mins/week), smoking status (current, previous, or never), drinking status (current, previous, or never), body

mass index (kg/m2), systolic blood pressure (mmHg), diastolic blood pressure (mmHg), uric acid

(mg/dL), C-reactive protein (mg/L), glycated hemoglobin (mmol/mol), low-density lipoprotein cholesterol

(mg/dL) and estimated glomerular filtration rate(mL/min/1.73m^2^). Abbreviations: TyG, triglyceride-glucose;

HR, hazard ratio; CI, confidence interval; COPD, chronic obstructive pulmonary disease; PD, panvascular

disease; Multimorbidity, multimorbidity of COPD and PD.

**Supplementary Table 4.3** Sensitivity Analysis: Excluding the Impact of COVID-19

| **Variable** | **TyG index** | | | | **P trend** | **Per 1 SD increase**  **in Log TyG values** |
| --- | --- | --- | --- | --- | --- | --- |
|  | **Quartile 1** | **Quartile 2** | **Quartile 3** | **Quartile 4** |  |  |
| Median | 8.07 | 8.49 | 8.85 | 9.35 |  |  |
| **COPD** |  |  |  |  |  |  |
| No. of cases/person-years | 645/938,380 | 579/933,810 | 1,032/932,203 | 1,170/932,503 | <0.001 |  |
| Model 1, HR (95%CI) | 1.00 | 1.35(1.21, 1.49) | 1.60(1.45, 1.77) | 1.82(1.65, 2.00) | <0.001 | 1.24(1.20, 1.28) |
| Model 2, HR (95%CI) | 1.00 | 1.10(0.99, 1.22) | 1.21(1.10, 1.34) | 1.31(1.19, 1.44) | <0.001 | 1.11(1.08, 1.15) |
| Model 3, HR (95%CI) | 1.00 | 1.06(0.96, 1.18) | 1.11(1.00, 1.23) | 1.12(1.00, 1.25) | 0.05 | 1.04(1.00, 1.08) |
| **PD** |  |  |  |  |  |  |
| No. of cases/person-years | 3,690/910,226 | 5,303/895,965 | 6,549/886,539 | 8,389/874,195 | <0.001 |  |
| Model 1, HR (95%CI) | 1.00 | 1.45(1.39, 1.52) | 1.81(1.74, 1.89) | 2.36(2.27, 2.45) | <0.001 | 1.37(1.35, 1.39) |
| Model 2, HR (95%CI) | 1.00 | 1.17(1.12, 1.22) | 1.33(1.27, 1.38) | 1.63(1.56, 1.69) | <0.001 | 1.22(1.20, 1.23) |
| Model 3, HR (95%CI) | 1.00 | 1.07(1.03, 1.12) | 1.12(1.07, 1.17) | 1.23(1.15, 1.32) | <0.001 | 1.09(1.05, 1.13) |
| **Multimorbidity** |  |  |  |  |  |  |
| No. of cases/person-years | 159/943,749 | 254/940,332 | 315/939,548 | 393/940,490 | <0.001 |  |
| Model 1, HR (95%CI) | 1.00 | 1.60(1.31, 1.95) | 1.98(1.64, 2.40) | 2.48(2.06, 2.98) | <0.001 | 1.38(1.30, 1.46) |
| Model 2, HR (95%CI) | 1.00 | 1.25(1.02, 1.52) | 1.39(1.15, 1.68) | 1.61(1.33, 1.93) | <0.001 | 1.20(1.13, 1.27) |
| Model 3, HR (95%CI) | 1.00 | 1.15(0.94, 1.41) | 1.17(0.96, 1.43) | 1.19(0.97, 1.46) | 0.15 | 1.06(0.98, 1.13) |

Data are presented as HR(95%CI) unless otherwise stated. The number of participants was 296,804 for

COPD, 293,641 for PD, and 296,750 for Multimorbidity after excluding individuals who developed the disease

after January 1, 2020, to exclude the impact of COVID-19. The TyG index was divided into 4 parts according

to quartiles, and the boundaries were 6.37~, 8.30~, 8.67~, 9.06~12.14. Model 1was an unadjusted model.

Model 2 was adjusted for age, gender (male or female), and Townsend Deprivation Index. Model 3 was

additionally adjusted for physical activity (<150 or ≥150 mins/week), smoking status (current, previous, or

never), drinking status (current, previous, or never), body mass index (kg/m2), systolic blood pressure

(mmHg), diastolic blood pressure (mmHg), uric acid(mg/dL), C-reactive protein (mg/L), glycated hemoglobin

(mmol/mol), low-density lipoprotein cholesterol(mg/dL) and estimated glomerular filtration

rate(mL/min/1.73m^2^).Abbreviations: TyG, triglyceride-glucose; HR, hazard ratio; CI, confidence interval;

COPD, chronic obstructive pulmonary disease; PD, panvascular disease; Multimorbidity, multimorbidity of

COPD and PD.

**Supplementary Table 4.4** Sensitivity Analysis: haemorrhagic stroke from the PD definition

| **Variable** | **TyG index** | | | | | | | | **P trend** | | **Per 1 SD increase in Log TyG values** | |
| --- | --- | --- | --- | --- | --- | --- | --- | --- | --- | --- | --- | --- |
|  | **Quartile 1** | | **Quartile 2** | | **Quartile 3** | | **Quartile 4** | |  | |  | |
| Median | 8.07 | | 8.49 | | 8.85 | | 9.35 | |  | |  | |
| **PD** |  | |  | |  | |  | |  | |  | |
| No. of cases/person-years | 3,964\922,833 | | 5,709/908,583 | | 7,140/898,764 | | 9,063/886,667 | | <0.001 | |  | |
| Model 1, HR (95%CI) | 1.00 | | 1.46(1.40-1.52) | | 1.85(1.78-1.92) | | 2.38(2.29-2.47) | | <0.001 | | 1.37(1.36-1.39) | |
| Model 2, HR (95%CI) | 1.00 | | 1.18(1.14-1.23) | | 1.36(1.31-1.41) | | 1.65(1.59-1.71) | | <0.001 | | 1.22(1.21-1.24) | |
| Model 3, HR (95%CI) | 1.00 | | 1.07(1.03-1.12) | | 1.14(1.09-1.19) | | 1.24(1.16-1.33) | | <0.001 | | 1.09(1.05-1.14) | |
| **Multimorbidity** |  |  | |  | |  | |  | |  | |  |
| No. of cases/person-years | 162/945,485 | 260/942,035 | | 330/941,275 | | 411/942,170 | | <0.001 | |  | |  |
| Model 1, HR (95%CI) | 1.00 | 1.61(1.32-1.96) | | 2.04(1.69-2.46) | | 2.54(2.12-3.05) | | <0.001 | | 1.38(1.31-1.46) | |  |
| Model 2, HR (95%CI) | 1.00 | 1.26(1.04-1.54) | | 1.44(1.19-1.74) | | 1.66(1.38-1.99) | | <0.001 | | 1.20(1.13-1.28) | |  |
| Model 3, HR (95%CI) | 1.00 | 1.15(0.95-1.41) | | 1.19(0.98-1.45) | | 1.21(0.98-1.48) | | 0.10 | | 1.05 (0.98-1.13) | |  |

Data are presented as HR(95%CI) unless otherwise stated. The number of participants was 297,285 after

excluding haemorrhagic stroke from the PD definition. The TyG index was divided into 4 parts according to

quartiles, and the boundaries were 6.37~, 8.30~, 8.67~, 9.06~12.14. Model1was an unadjusted model. Model 2

was adjusted for age, gender (male or female), and Townsend Deprivation Index. Model 3 was additionally

adjusted for physical activity (<150 or ≥150 mins/week), smoking status (current, previous, or never),

drinking status (current, previous, or never), body mass index (kg/m2), systolic blood pressure (mmHg),

diastolic blood pressure (mmHg), uric acid(mg/dL), C-reactive protein (mg/L), glycated hemoglobin

(mmol/mol), low-density lipoprotein cholesterol(mg/dL) and estimated glomerular filtration

rate(mL/min/1.73m^2^). Abbreviations: TyG, triglyceride-glucose; HR, hazard ratio; CI, confidence

interval; COPD, PD, panvascular disease; Multimorbidity, multimorbidity of chronic obstructive pulmonary

disease and PD.

**Supplementary Table 5** variance inflation factor (VIF) values for covariates in the fully adjusted models.

| **Variable** | **VIF^a^** |
| --- | --- |
| Female | 1.538 |
| Age, years | 1.615 |
| Townsend Deprivation Index | 1.059 |
| Physical activity, min/week | 1.008 |
| Smoking status | 1.025^b^ |
| Drinking status | 1.011^b^ |
| BMI, kg/m^2^ | 1.421 |
| Uric acid, mg/dL | 1.902 |
| C-reactive protein, mg/L | 1.084 |
| Glycated hemoglobin, mmol/mol | 1.119 |
| Systolic blood pressure, mm Hg | 2.301 |
| Diastolic blood pressure, mm Hg | 2.229 |
| Low-density lipoprotein cholesterol, mg/dL | 1.126 |
| eGFR, mL/min/1.73 m^2^ | 1.595 |
| TyG index (quartile) | 1.056^b^ |

a VIF values were averaged across five imputed datasets. All values < 5, indicating no problematic multicollinearity.

b Multi-category variable (df > 1); $\text{GVIF}^{1/\left( 2\cdot\text{df} \right)}$ is reported, directly comparable to $\sqrt{\text{VIF}}$; threshold < 2.24.

Abbreviations: TyG, triglyceride-glucose; eGFR, estimated glomerular filtration rate.
